# An interlocked oscillator model for high-frequency firing of the midbrain dopaminergic neuron

**DOI:** 10.1186/1471-2202-12-S1-O19

**Published:** 2011-07-18

**Authors:** Alexey Kuznetsov, Joon Ha

**Affiliations:** 1 Department of Mathematical Sciences and Center for Mathematical Biosciences, Indiana University, USA; 2 Purdue University Indianapolis, Indianapolis, IN 46202, USA; 3 NIDDK, NIH, Bethesda, MA 20892, USA

## 

**Figure 1:**
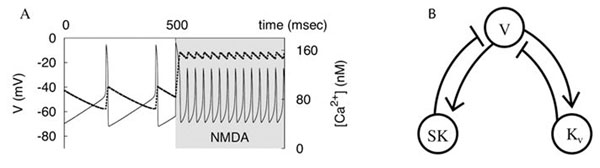
**(A)** The model switches to high-frequency oscillations at the onset of NMDA receptor stimulation at 500 msec. Dashed is the Ca^2+^ concentration; Solid is the voltage. The increase in the frequency is based on the reduction in the amplitude of Ca^2+^ oscillations. **(B)** The structure of the model. The SK-type Ca2+-dependent potassium current and the putative voltage-dependent potassium current create two negative feedback loops. The loops are interlocked by the voltage variable.

Dopamine neurotransmission has been found to play a role in addictive behavior and is impaired in psychiatric disorders. Dopaminergic (DA) neurons display two functionally distinct modes of electrophysiological activity: low- and high-frequency firing. The puzzling feature of the DA neuron is the combination of its high-frequency response to N-methyl-D-aspartate (NMDA) receptor activation coupled with the inability of other treatments to elevate its frequency effectively. We suggest a new computational model that reproduces this combination of responses and accounts for recent experimental data. The model is presented in two morphologies: (1) a reconstruction of a DA neuron and (2) a single compartment that ignores the spatial structure of the neuron. We show that these two model morphologies display very similar patterns. Therefore, an equipotential representation of the DA neuron is sufficient for combining its high- and low-frequency firing. Our comparison of the reconstructed morphology and the one-compartment model suggests that different regions of the neuron contribute differently to the high- and low frequencies. The model suggests how NMDA current restricted to the soma evokes high-frequency oscillations (Fig. 1A) - a recent experimental result. Alternatively, distal NMDA stimulation must span an extensive part of the dendritic tree to evoke the burst. The two distinct patterns of stimulation suggest that the burst may report different cue types, such as saliency and reward. In both cases, the voltage dependence of the NMDA current is central for this capability. Additionally, we introduced a putative potassium current that allows for sustained oscillations under blockade of the calcium-dependent (SK-type) potassium current. Given multiple de- and repolarizing currents that sustain pacemaking, the neuron has two interlocked mechanisms (calcium-dependent and independent; Fig. 1B) for producing oscillatory activity.

